# Inclusion of live yeast and mannan-oligosaccharides in high grain-based diets for sheep: Ruminal parameters, inflammatory response and rumen morphology

**DOI:** 10.1371/journal.pone.0193313

**Published:** 2018-02-21

**Authors:** Tatiana Garcia Diaz, Antonio Ferriani Branco, Fernando Alberto Jacovaci, Clóves Cabreira Jobim, Dheyme Cristina Bolson, João Luiz Pratti Daniel

**Affiliations:** Department of Animal Science, State University of Maringá; Av. Colombo, Bloco J45, Maringá, PR, Brazil; CNR, ITALY

## Abstract

The objective of this study was to evaluate the effects of dietary supplementation with live yeast (*Saccharomyces cerevisiae*), mannan-oligosaccharides and the combination of these additives on the inflammatory response, ruminal parameters and rumen morphology of sheep fed a high grain-based diet. Thirty-Two Dorper x Santa Ines crossbred lambs with an average weight of 24±2 kg were distributed in a completely randomized design. The animals were housed in individual stalls and fed ad libitum. Diet treatments were: Control (without additive); LY (2 g/kg DM of live yeast, *Saccharomyces cerevisiae*), MOS (2 g/kg DM of mannan-oligosaccharides) and LY+MOS (2 g/kg DM of LY + 2 g/kg DM of MOS). The experiment lasted 42 days. The supplementation with MOS alone and the additives combination resulted in increased ruminal pH (P<0.01), while the total concentrations of short chain fatty acids (SCFA) in the rumen were higher (P<0.05) only in the diets with LY and MOS. Ammonia (NH_3_) concentration in the rumen decreased (P<0.04) with the additives usage. Diets with LY, MOS and with additives combination reduced (P<0.01) the levels of lipopolysaccharides (LPS) in the plasma with values of 0.46; 0.44 and 0.04 EU/mL, respectively when compared to the control (0.93 EU/mL). MOS and LY+MOS treatments had reduced stratum corneum thickness (P<0.01) in comparison to the control treatment. The total thickness of ruminal epithelium was lower with the addition of MOS in the diet (P<0.03) than with LY additive. The incidence and severity of hepatic abscesses in animals whose diet was supplemented with LY and LY+MOS was lower (P<0.05) than in animals fed the control diet. The use of LY, MOS and LY+MOS in the high-concentrate diets for sheep reduced NH_3_ concentrations and LPS translocation into the bloodstream. Diets containing MOS and LY+MOS enhanced the health of the ruminal epithelium by reducing the thickness of the stratum corneum, and diets containing LY and LY+MOS decreased the incidence and severity of hepatic abscesses.

## Introduction

The use of high-concentrate diets, mainly grains, is an increasingly common practice in ruminant feeding to improve milk production and body weight. However, this type of diet contains carbohydrates that are rapidly fermentable in the rumen and increases the risk of metabolic disorders such as ruminal acidosis, foot problems and hepatic abscesses [1, 2]. This condition develops mainly due to an excessive accumulation of organic acids in the rumen, which causes a drop in ruminal pH (<5.8) and lesions in the gastrointestinal barrier [3, 4]. Ruminal acidity leads to the death of gram-negative bacteria with the corresponding release of endotoxins (lipopolysaccharides, LPS), activating a cascade of inflammatory mediators, and affecting the animal productive performance [5, 6]. For this reason, the economic and health consequences of subacute ruminal acidosis can be considered one of the most important problems in animal feedlot systems.

Subacute ruminal acidosis is difficult to diagnose, which makes it hard to treat and, in most cases, the treatment is not very effective. Preventive measures should be mainly based on feeding management. Thus, one of the strategies is the dietary inclusion of probiotics, such as live yeast cultures, and prebiotics, such as mannan-oligosaccharides (MOS), complex carbohydrate molecules, mannose (mannoproteins), β-glucans and proteins derived from the outer cell wall of *Saccharomyces cerevisiae* yeast, that have been identified as moderators of the ruminant immune system [7, 8]. The possible effects of these additives on the digestive tract microbiota, ruminal pH regulation, and immunostimulation are beneficial to digestibility, performance, and health of animals [9, 10].

The combined use of prebiotics and probiotics in animal feeds is advantageous to potentiate the animal response and constitutes a new concept in additives usage. This combination is a plausible alternative to improve herd health through synergistic effects, consequently, promoting animal performance and nutritional status [11].

Certainly, there is evidence of the favorable use of live yeast and MOS in ruminant (cattle and sheep) production [10], although few studies have been conducted to evaluate the effects of MOS and combined effects on ruminal fermentation, digestibility, and ruminant immune system.

The present study was carried out to evaluate the effects of dietary supplementation with LY, MOS and the combination of these additives (LY+ MOS) on the inflammatory response, ruminal morphology, and incidence of hepatic abscesses in sheep fed a grain-based diet.

## Material and methods

All procedures performed in accordance with Brazil’s National Council for the Control of Animal Experimentation (CONCEA) guidelines and were authorized by the Ethics Committee for Animal Use of the State University of Maringá, PR, Brazil (Protocol number: 6479060416-CEUA/UEM). The experimental period was conducted from March to May 2015.

### Animals, experimental design and treatments

The animals used in this experiment, thirty-two Dorper x Santa Inês crossbred lambs with a mean body weight of 24±2 kg, were distributed in a completely randomized experimental design with four treatments and eight replicates. Dietary treatments were: Control: without additive; LY: 2 g/kg DM of live yeast (*Saccharomyces cerevisiae* 10^10^ cfu/g; Procreatin-7®, LeSaffre Feed Additives, Brazil); MOS: 2 g/kg DM of MOS (460 g/kg mannan-oligosaccharides, yeast cell wall; Safmannan® LeSaffre Feed Additives, Brazil); LY+MOS: 2 g/kg DM of live yeast (*Saccharomyces cerevisiae* 10^10^ cfu/g) + 2 g/kg DM of MOS (460 g/kg yeast cell wall). The animals were housed in 0.60×0.90 m individual stalls with a slatted floor in a masonry shed and equipped with individual feeders and drinkers. They were dewormed and vaccinated against clostridium using the Sintoxan® Polyvalent vaccine, Merial, Brazil.

The experimental diet is shown in Table 1.

**Table 1:** Composition of experimental diets (g/kg of dry matter).

Item	Treatments			
	Control	LY^1^	MOS^2^	LY+ MOS^3^
Corn Silage	90.0	90.0	90.0	90.0
Whole corn	765.0	765.0	765.0	765.0
Concentrate				
Soybean meal	100.0	100.0	100.0	100.0
Limestone	20.0	20.0	20.0	20.0
Common salt	3.0	3.0	3.0	3.0
Urea	7.0	7.0	7.0	7.0
Ammonium chloride	5.0	5.0	5.0	5.0
Premix	10.0	10.0	10.0	10.0
*Saccharomyces cerevisiae*, LY	-	2.0	-	2.0
Mannan-oligosaccharides, MOS	-	-	2.0	2.0
Fine ground corn, mg/g	9.990	7.980	7.980	6.030
Sodium selenite, mg/g	0.002	0.002	0.002	0.002
Cobalt sulfate, mg/g	0.008	0.008	0.008	0.008
Calcium Iodate, mg/g	0.008	0.008	0.008	0.008
Chemical composition				
DM, g/kg as fed	770.0			
CP	140.0			
NDFap	175.0			
ADF	075.5			
NFC	603.0			


^1^LY: live yeast (*Saccharomyces cerevisiae)*

^2^MOS: mannan-oligosaccharides

^3^LY+MOS: live yeast (*Saccharomyces cerevisiae) +* MOS.

### Experimental procedures

The animals were fed twice a day at 08h00min and 16h00min. Diets were offered in a sufficient amount, allowing for 10% leftovers which were collected daily from the feeders and weighed for individual consumption adjustment. The leftover samples were frozen at -20°C and thawed at the end of the collection period. Next, they were homogenized and pre-dried in a forced circulation oven at 55°C for 72 h. Subsequently, the samples were ground in a Wiley mill equipped with a 1 mm sieve and mixed in the same ratio (10% of sample based on the dry weight) to form samples composed of leftovers per animal per treatment.

After the second weighing at the 42nd experimental day, the animals were selected to be slaughtered on a weekly basis, in groups of 8 animals (2 animals per treatment) with body weight between 37 and 40 kg. Blood samples of 15 mL were drawn by jugular puncture using vacutainer tubes with sodium heparin after 16 h of fasting. The blood was centrifuged at 3000 x *g* for 15 min to obtain plasma and stored in Eppendorf tubes at -20°C for LPS analysis, according to Khafipour et al. [6], serum amyloid A (SAA) and haptoglobin (Hp).

The slaughter followed the regulations for inspection of industrial sanitation for products of animal origin, RIISPOA [12]. They were transported to the abattoir by truck. The slaughterhouse was located within 600 m from the stalls. Immediately after their arrival, the animals were electrically stunned and slaughtered by throat cut. The knife used for cattle has a long, extremely sharp, and undamaged blade. The intention was to produce the immediate outpouring of blood by severing both jugular veins and both carotid arteries. After bleeding and removal of the skin, the opening was made along the entire length of the ventral midline of the abdomen to eviscerate. The head and legs were separated from the carcass. During the slaughter, rumen and small intestine were set aside for the collection of ruminal contents and intestinal digesta. Rumen content was filtered through four layers of gauze and had pH readings measured with a digital potentiometer according to Ding et al. [9]. For assessment of NH_3_ concentration, a 50-mL ruminal fluid sample was acidified with 1 mL sulfuric acid (1:1 ratio), while for determination of SCFA another 50-mL sample was used. After these procedures, the specimens were stored at -20°C until further analyses. After slaughter the carcasses were placed at room temperature (18°C) for 6 h, then in a refrigerated room set to 4°C until 24 h postmortem.

Lipopolysaccharides concentration was quantified from samples of 15 mL ruminal fluid and 15 mL intestinal digesta as well. They were transferred into sterile tubes and centrifuged at 6000 x *g* for 15 min at 4°C. The supernatant was stored at -20°C for analysis of LPS concentration, as described by Khafipour et al. [6] and Emmanuel et al. [13].

### Chemical analysis

Feed samples used in the experimental diets and the leftovers were analyzed according to AOAC [14] for dry matter (DM; 934.01), ash (942.05), crude protein (CP; 984.13), and ether extract (EE; 920.39). The analyses of acid detergent fiber (ADF) [15], neutral detergent fiber (NDF) [16], and NDF corrected for ash and protein (NDFap) were obtained by using neutral detergent insoluble nitrogen (NDIN) (without sodium sulfite (Na_2_SO_3_) and with added thermostable α-amylase). The non-fibrous carbohydrate (NFC) contents were estimated using the NRC equation [17].

The SAA and Hp were determined with the commercial kits, Tridelta SAA Multispecies Assay and Tridelta PHASE Haptoglobin Multispecies Assay, respectively. The analytical sensitivities of these tests in serum have been determined as 1.5 μg/mL for SAA and 0.05 μg/mL for Hp by the manufacturer. Plasma LPS analysis, ruminal fluid and duodenal digesta were evaluated with the commercial kit, ToxinSensor ™ chromogenic LAL Endotoxin Assay, GenScript. The minimum detection level of LPS in the plasma was 0.01 EU/mL.

Ammonia (NH_3_) concentration was measured through the technique of Chaney and Marbach [18]; while the concentration of SCFA was determined by using a gas chromatograph (SHIMADZU, model GC-2014) equipped with an auto injector (model AOC-20). The injector reached a temperature of 200°C, and the column temperature was 80°C/3min till 240°C (20 degrees/min). The column used was HP INNOwax - 19091N (30m long, 0.32mm ID, 0.50 μm film) and the detector was flame ionization.

### Ruminal histology

**Fig 1:**
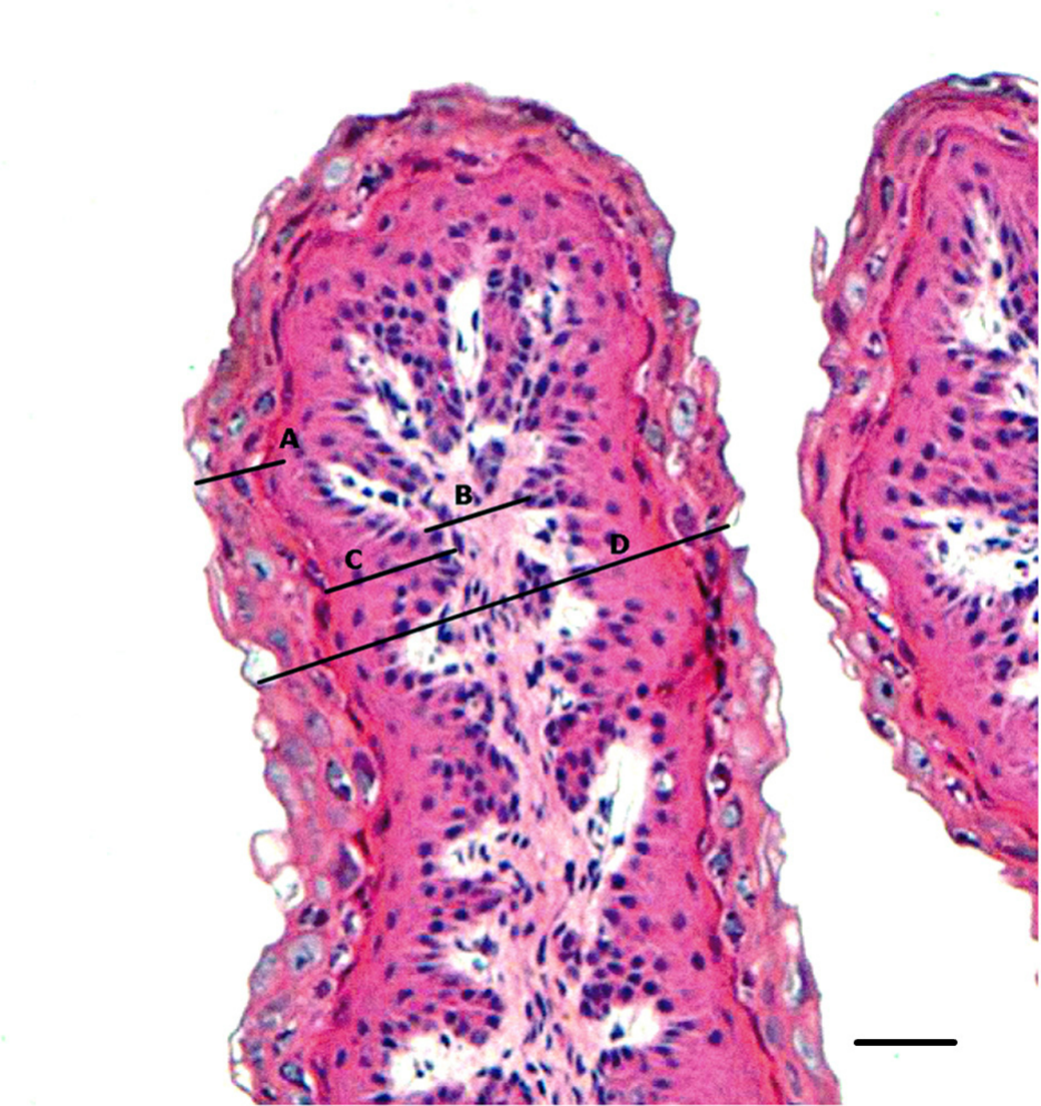
Histology of ruminal papillae of sheep fed grain-based diets stained by hematoxylin-eosin method using the 10x objective. (A) Stratum corneum thickness (B) Connective tissue width. (C) Non-keratinized epithelium (D) Total epithelium thickness.

After digesta removal and rumen lavage with distilled water, a fragment of approximately 1 cm^2^ was collected from the ventral sac region of the rumen. Ruminal tissue samples were washed in phosphate buffer solution (0.1 M, pH 7.4) and fixed in 10% buffered formalin solution. The histological sections were mounted on slides and stained by hematoxylin-eosin techniques according to the methodology proposed by Prophet et al. [19]. The microscopic variables evaluated were papillae width, connective tissue width, total epithelium thickness, and stratum corneum thickness. The thickness of the nonkeratinized layer was calculated by the difference between the total thickness of the epithelium and the thickness of the stratum corneum. The images and analyses were performed using an Olympus BX41 microscope coupled to a computer, through Motic imaging software plus 2.0 ML (Fig 1).

### Hepatic abscesses

Hepatic abscesses (HA) severity was classified based on a scale of 0, 1, 2 and 3 [20], and categorized as follows: (0) liver without abscesses; (A- = 1) liver with one or two small abscesses (smaller than 2.5 cm in diameter) or abscess scars; (A = 2) liver with two to four active abscesses (smaller than 2.5 cm in diameter); (A + = 3) liver with one or more large abscesses (larger than 2.5 cm in diameter) and portions of the diaphragm adhered to the liver surface. The incidence of hepatic abscesses was calculated by considering the percentage of animals identified with abscesses within each treatment.

### Statistical analysis

Data were analyzed using the GLM procedure of the SAS statistical package [21]. The means among the treatments were compared by the Tukey test at α = 0.05 probability. The statistical model used was as follows:

Yij=μ+Ti+eij

Yij = observed value of the variables studied relative to each individual *j* receiving a treatment *i*; μ = general constant; T*i* = treatment effect *i*, with *i* ranging from 1 to 4; e*ij* = random error associated with each observation. The abscess frequencies were calculated by the FREQ procedure. The incidence and severity of abscesses were analyzed by the SAS Logistic procedure [21], using the chi-square test and adopting α = 0.05.

## Results and discussion

### Rumen parameters

Rumen pH values obtained during the slaughter were low in all treatments (Table 2) after 16 hours of fasting (as measured), suggesting a possible subacute ruminal acidosis. Episodes of this metabolic disorder are diagnosed when the rumen pH drops to a suboptimal level (pH<5.8) for a period longer than 5.4 h per day [3, 22].

**Table 2:** Values of ruminal pH, ammonia (NH_3_) and short chain fatty acids (SCFA) of sheep fed high concentrate diets supplemented with live yeast (LY, *Saccharomyces cerevisiae)*, mannan-oligosaccharides (MOS) and additives combination (LY+ MOS).

Item	Treatments				SEM^1^	P
	Control	LY	MOS	LY+MOS		
Ruminal pH	5.18^c^	5.30b^c^	5.34^b^	5.50^a^	0.07	0.01
NH_3_ (mg/dL)	36.54^a^	29.45^b^	24.93^c^	28.14^c^	1.62	0.04
Total SCFA, mM	136.9^c^	157.4^a^	145.4^b^	132.3^c^	3.57	0.05
SCFA						
Acetate (A)	68.68	77.60	69.83	61.55	4.9	0.12
Propionate (P)	49.78	57.37	60.46	52.17	2.2	0.29
Butyrate (B)	14.27	18.78	11.37	14.68	1.5	0.39
Isobutyrate	0.40	0.42	0.42	0.43	0.04	0.99
Isovalerate	1.13	1.56	1.06	1.68	0.18	0.57
Valerate	2.67	1.66	2.67	1.77	0.18	0.18
Proportion A: P	1.54	1.44	1.66	1.18	0.08	0.28

^1^SEM = standard error mean; Means followed by different letters in the same row are statistically different according to the Tukey test (α = 0.05).

Treatments with MOS and LY+MOS had higher (P<0.01) ruminal pH values than the control treatment. However, the pH of the animals fed only LY was similar (P>0.05) to those that received the control treatment (Table 2).

Ding et al. [9], Silberberg et al. [10], Chung et al. [23], and Vyas et al. [24] stated that LY and MOS increase the ruminal pH of animals fed high-grain diets and reduce drastic variations in pH values, resulting in greater stability of the ruminal environment. The use of LY *in vitro* studies has demonstrated that these additives provide dicarboxylic acids, particularly malic acid, pro-vitamins, and micronutrients, favoring the growth and activity of cellulolytic bacteria, lactic acid bacteria and ruminal protozoa [23]. The colonization of lactate utilizers (*Megasphaera elsdenii and Selenomonas ruminantium*) with the use of LY was also verified by Pinloche et al. [25] in Holstein cows. Possibly, the combination of LY+MOS (probiotic and prebiotic) in the diets may have generated a synergistic effect that favored ruminal pH increase. Although the pH increased with the addition of LY+MOS into the diet, this result does not indicate that there was a stabilizing effect on ruminal pH, because the highest ruminal pH (5.50) was below 5.8, the level considered to be indicative of ruminal acidosis [3].

Supplementations with LY and MOS increased (P<0.05) the total concentration of SCFA (Table 2). Similar results were reported by Li et al. [8], who also found increased production of SCFA using increasing levels of oligosaccharides (0.2 to 0.8%) in the basal diet of sheep. According to Pinloche et al. [25], a rise in SCFA concentration may be related to increased metabolic activity by ruminal microorganisms, and stimulation of microbial growth, which is beneficial for the maintenance and production of the ruminant [26]. However, the accumulation and dissociation of SCFA in ruminal fluid lowers the pH, as observed in this experiment, which may cause morphological and histological changes in the ruminal papillae and thus impair the barrier function of the ruminal epithelium [27, 28, 29]. It is important to note that the production of SCFA, like the pH, presents variations throughout the day and in relation to the feeding periods, and more evaluations are necessary to verify the possible protective effects of LY and MOS.

Ammonia (NH_3_) concentrations were lower (P<0.04) in all treatments with additives when compared to control treatment. This result is possibly due to a greater flow of microbial protein into the small intestine of the animals and, consequently, promoting a better efficiency of nitrogen uptake (Table 2). Similarly, Alshaikh et al. [30] reported reductions in NH_3_ levels by adding LY culture to concentrate-based diets. More recently, Vyas et al. [24] observed no effect of dietary supplementation with live yeast or inactive dry yeast on the ruminal NH_3_ concentration of beef heifers, which suggests that results are still controversial about this parameter.

### Lipopolysaccharides and acute-phase proteins

The concentrations of free LPS in the plasma and ruminal fluid measured in this study (Table 3) were higher than the values obtained by Huo et al. [2] in goats fed diets with low concentrate rates. This result may be related to the cellular lysis of ruminal bacteria sensitive to low pH as a consequence of the high-grain diet [4, 6, 13].

**Table 3:** Lipopolysaccharides (LPS), serum amyloid A (SAA) and haptoglobin (Hp) in sheep fed grain-based diets supplemented with live yeast (LY, *Saccharomyces cerevisiae*), mannan-oligosaccharides (MOS) and the combination of these additives (LY+MOS).

Item	Treatment				SEM^2^	P
	Control	LY	MOS	LY+MOS		
LPS, EU^1^/mL						
Plasma	0.94^a^	0.46^b^	0.44^b^	0.04^c^	0.02	0.01
Ruminal fluid	46,132	44,594	42,480	43,276	1,953	0.92
Duodenal fluid	45,473	45,292	43,075	43,544	1,860	0.95
SAA (μg/mL)	28.16	29.63	28.64	29.14	3.49	0.75
Hp (μg/mL)	316.37	316.29	317.11	318.19	4.06	0.46

^1^EU: Endotoxins Unit

^2^SEM = standard error mean; Means followed by different letters in the same row are statistically different according to the Tukey test (α = 0.05).

Concentrations of LPS achieved here were like those reported by Khafipour et al. [6] who obtained increased LPS concentration in peripheral blood by replacing only 21% DM of the total diet of dairy cows for a diet mixed with pellets comprised of 50% wheat and 50% barley. On the other hand, Lei et al. [31] fed beef cattle a 60% concentrate diet to induce subacute ruminal acidosis and obtained rises in free LPS concentrations of 44,006 EU/mL and 0.96 EU/mL in the ruminal fluid and plasma, respectively.

Lipopolysaccharides (LPS) translocation into the systemic circulation may occur because of increased permeability of the rumen epithelium [5], which allows bacteria and bacterial toxins (LPS) to enter the portal circulation, causing metabolic changes such as liver abscesses and an inflammatory response [1, 4].

Supplementation with LY, MOS, and LY+MOS resulted in lower plasma concentrations of LPS (P<0.01). The treatment with LY+ MOS had the lowest concentration of LPS (0.04 EU/mL; Table 3), probably because of a synergistic effect with the use of both additives. This synergism may occur due to an effectively binding driven by electrostatic forces. In this case, the yeast cell wall and MOS contain positively charged ligands capable of adsorbing endotoxins by the interaction with a negatively charged group (phosphate). This binding enables the elimination of LPS from the animal digestive tract, preventing its adsorption into the bloodstream [31].

The concentrations of acute phase proteins, SAA and Hp (Table 3) were high, possibly [32, 33] because of the LPS presence in the bloodstream. LPS activates the release of proinflammatory cytokines (tumor necrosis factor-α, interleukins-1 and 6) by hepatic macrophages that stimulate the release of SAA and Hp proteins as an inflammatory response [6]. The acute phase response has the function of preventing additional injury, isolating and destroying infectious organisms, and activating the repair processes necessary to return the organism to normal function [34]. The concentrations of acute-phase proteins were not influenced by the addition of LY (*Saccharomyces cerevisiae*), MOS, or their combination (LY+MOS) in grain-based diets for sheep (P>0.05; Table 3).

### Rumen histology

The thickness of the stratum corneum normally increases due to the response of the ruminal epithelium to a high content of non-fibrous carbohydrates in the diet [28]. Under the conditions used in this experiment (76.5% whole corn; 60.3% NFC), feeding with the control and LY diets resulted in increased (P<0.05) stratum corneum thickness (23.66 and 22.14 μm, respectively) compared with the MOS and LY+MOS diets (16.51 and 19.36 μm, respectively, Table 4). The stratum corneum thickness observed with the MOS and LY+MOS diets can be considered normal for forage-based diets (17.3 μm), according to Steele et al. [35]. These results suggest that MOS and LY+MOS have a protective effect on the ruminal epithelium against the damage caused by severe ruminal acidosis that occurs when feeding grain-based diets. The protection mechanism of these additives seems to be related to stabilization of the rumen pH, which may reduce the length of time during which the pH is below 5.8 [23, 24, 36]. However, the evidence provided in this study is currently insufficient to demonstrate the effect, and more research is needed for a definitive conclusion.

**Table 4:** Morphological parameters of rumen papillae of sheep fed a grain-based diet supplemented with live yeast (LY, *Saccharomyces cerevisiae*), mannan-oligosaccharides (MOS), and the combination of these additives (LY + MOS).

Item, μm	Treatment				SEM^1^	P
	Control	LY	MOS	LY+MOS		
Papillae total width	288.80	296.50	279.90	283.20	3.44	0.16
Connective tissue width	53.07	59.10	55.32	54.19	2.76	0.62
Epithelium thickness	80.85^ab^	87.26^a^	73.81^b^	80.52^ab^	2.79	0.03
Stratum corneum thickness	23.66^a^	22.14^a^	16.51^c^	19.36^b^	0.63	0.01
Nonkeratinized thickness	57.19	65.12	57.29	66.72	1.62	0.26

^1^SEM = standard error mean; Means followed by different letters in the same row are statistically different according to the Tukey test (α = 0.05).

The stratum corneum has a large amount of keratin in the cytoplasm and few cellular organelles that act as a physical barrier and reduce the transport of SCFA to the deepest layers of the epithelium [37]. Although the stratum corneum was thicker in the control and LY treatments (P<0.05), the thickness of the nonkeratinized layer was not affected by the treatments (P>0.05). The additives did not influence (P>0.05) the widths of the papillae, connective tissue or nonkeratinized layer (Table 4).

### Hepatic abscesses

Hepatic abscesses may develop because of lesions on ruminal epithelium (ruminitis) caused by a high consumption of rapidly fermentable feeds [38, 39]. When the integrity of ruminal papillae is damaged, bacteria that are usually part of the normal ruminal microbiota such as *Fusobacterium necrophorum* and *Actinomyces pyogenes*, enter the portal circulation and consequently, into the liver, leading to infections establishment and promoting the *parakeratosis-rumenitis liver abscesses complex* [40, 41].

The incidence and severity (described as the percentage of livers exhibiting grades A and A +) of hepatic abscesses in animals fed the LY and LY+MOS diets was lower (P<0.05) than in animals fed the control and MOS diets (Table 5). In the control diet group, two animals had hepatic abscesses of type A + (larger than 2.5 cm in diameter). It is very important to search for efficient additives to prevent this type of problem in grain-based diets, since these abscesses can decrease the carcass weight by 11.6%, the yield by 2.52%, and cause liver adhesion to the carcass, resulting in liver failure [42].

**Table 5:** Incidence and severity of hepatic abscesses in sheep fed grain-based diets supplemented with live yeast (LY, *Saccharomyces cerevisiae*), mannan-oligosaccharides (MOS), and additives combination (LY+ MOS).

Item	Treatments				SEM^1^	P
	Control	LY	MOS	LY+ MOS		
0 (No abscesses), n	2	5	3	5	-	-
A-, n	2	2	3	2	-	-
A, n	2	1	1	1	-	-
A+, n	2	0	1	0	-	-
Abscesses incidence, %	75.0^a^	37.5^b^	62.5^a^	37.5^b^	14.5	0.01
Abscesses severity, %^2^	50.0^a^	12.5^b^	25.0^a^	12.5^b^	13.0	0.02

^1^SEM = standard error mean; (0) liver without abscesses; (A- = 1) liver with one or two small abscesses (smaller than 2.5 cm in diameter) or abscess scars; (A = 2) liver with two to four active abscesses (smaller than 2.5 cm in diameter); (A + = 3) liver with one or more large abscesses (larger than 2.5 cm in diameter) and portions of the diaphragm adhered to the liver surface. ^2^Abscesses severity, % of livers within A and A+ classification (more harmful); Means followed by different letters in the same row are statistically different (α = 0.05).

Although the group fed the MOS diet did not differ from the control diet group in relation to the incidence and severity of liver abscesses, the severity of the abscesses in this group was half that of the animals fed the control diet (25 vs. 50%, Table 5). The mannoproteins present in the MOS additive have a high binding affinity for Type 1 fimbriae lectins on the surface of pathogenic bacteria. This mechanism allows MOS to bind to a large variety of microorganisms in a competitive way, blocking colonization by these pathogens [43]. However, these data were obtained from *in vitro* studies. More *in vivo* studies should be performed to corroborate these results, since this kind of mechanism has been little discussed in previous literature.

## Conclusion

The supplementations of 2 g/kg of DM of LY (*Saccharomyces cerevisiae*, 10^10^ cfu/g), 2 g/kg of DM of MOS (460 g/kg of *Saccharomyces cerevisiae* cell wall), and the combination of these additives in high grain-based diets for sheep reduced NH_3_ concentrations and LPS translocation into the bloodstream. The MOS and LY+MOS enhanced the health of the ruminal epithelium by reducing the stratum corneum thickness.

Live yeast (LY) and the additives combination (LY+MOS) reduced the incidence of hepatic abscesses in animals fed high grain-based diets. The combination of LY+MOS is a plausible strategy to attenuate the effects acidosis of finishing lambs fed diets with high grain content, but more studies are required to confirm these results.

## Supporting information

S1 Fig Ruminal pH of sheep fed high concentrate diets supplemented with live yeast (LY, *Saccharomyces cerevisiae)*, mannan-oligosaccharides (MOS) and additives combination (LY+ MOS). (XLSX)

S2 Fig Ammonia (NH_3_) of sheep fed high concentrate diets supplemented with live yeast (LY, *Saccharomyces cerevisiae)*, mannan-oligosaccharides (MOS) and additives combination (LY+ MOS). (XLSX)

S3 Fig Histology of ruminal papillae of sheep fed grain-based diets stained by hematoxylin-eosin method. a) Control; b) Yeast, c) MOS d) Yeast+ MOS.
(TIF)
