# Correction: Inclusion of live yeast and mannan-oligosaccharides in high grain-based diets for sheep: Ruminal parameters, inflammatory response and rumen morphology

**DOI:** 10.1371/journal.pone.0196184

**Published:** 2018-04-17

**Authors:** Tatiana Garcia Diaz, Antonio Ferriani Branco, Fernando Alberto Jacovaci, Clóves Cabreira Jobim, Dheyme Cristina Bolson, João Luiz Pratti Daniel

There is an error in the second sentence under the “Animals, experimental design and treatments” subheading in the Methods and Materials section. The correct sentence is: Dietary treatments were: Control: without additive; LY: 2 g/kg DM of live yeast (Saccharomyces cerevisiae 10^10^ cfu/g; Procreatin-7®, Phileo LeSaffre Animal Care); MOS: 2 g/kg DM of MOS (460 g/kg mannan-oligosaccharides, yeast cell wall; Safmannan®, Phileo LeSaffre Animal Care); LY+MOS: 2 g/kg DM of live yeast (Saccharomyces cerevisiae 10^10^ cfu/g) + 2 g/kg DM of MOS (460 g/kg yeast cell wall).
