# Supplementary figures and images for: Inclusion of live yeast and mannan-oligosaccharides in high grain-based diets for sheep: Ruminal parameters, inflammatory response and rumen morphology
Source: PLoS One. 2018 Feb 21;13(2):e0193313. doi: 10.1371/journal.pone.0193313 (PMC5821403; doi:10.1371/journal.pone.0193313)

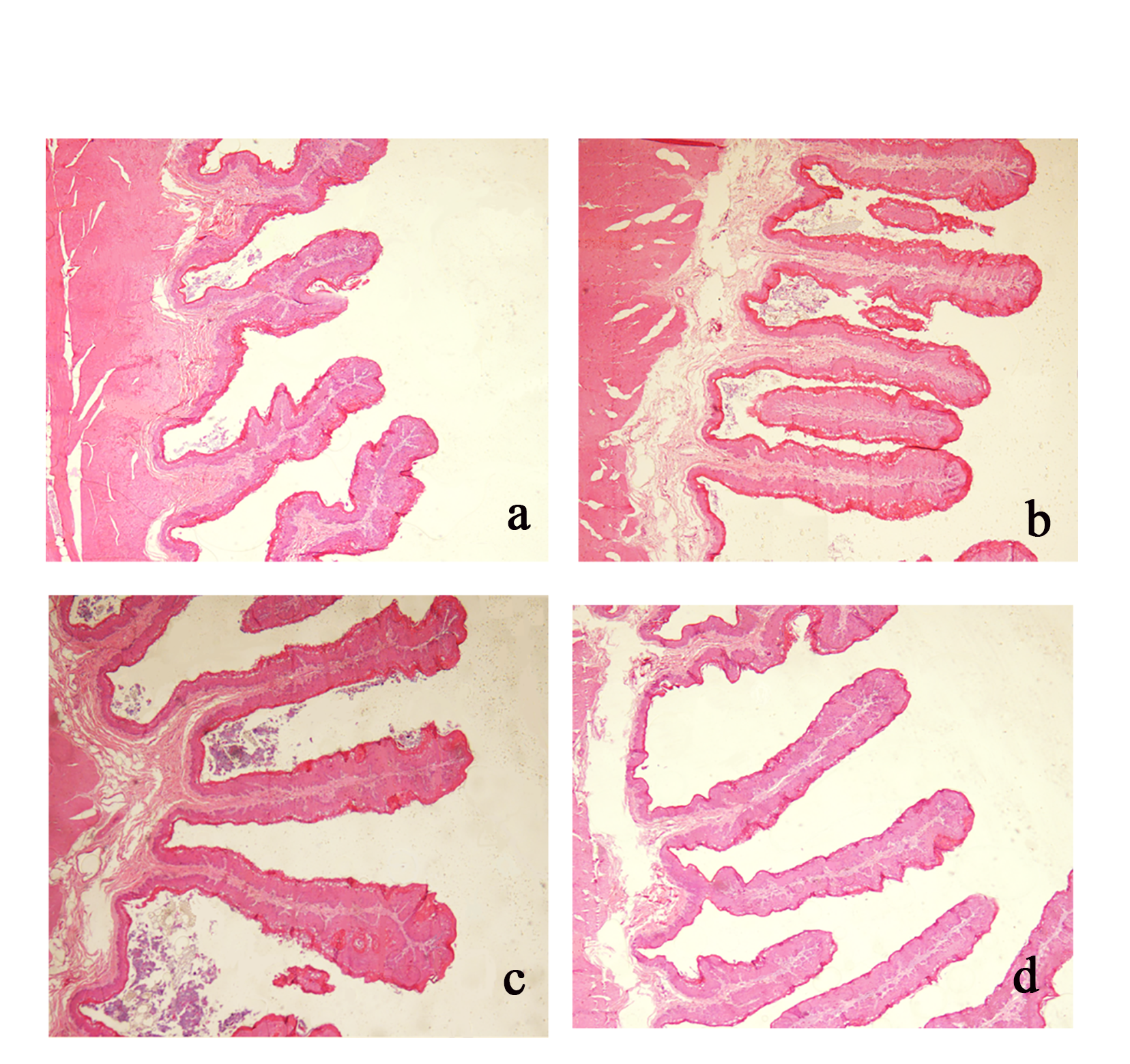

Supplement: S3 Fig — a) Control; b) Yeast, c) MOS d) Yeast+ MOS. (TIF) [file pone.0193313.s003.tif]
